# menting Data Governance with Multi-Modal Privacy-Preserving Record Linkages between Restricted and Public Open Enclaves

**DOI:** 10.23889/ijpds.v9i5.2898

**Published:** 2024-09-18

**Authors:** Kenneth Gersing, Sam Michael, Fred Prior, Joel Saltz, Richard Moffitt, Sara Rogovin, Jasmin Phua, Shaun Grannis, Ben Amor, Andrew Girvin, Ahmad Baghal

**Affiliations:** 1 National Institutes of Health; 2 University of Arkansas Medical Sciences; 3 Stony Brook University; 4 Emory University; 5 Datavant; 6 Regenstrief Institute; 7 Palantir Technologies

Data centralization is increasingly non-viable in the changing landscape of data privacy and data governance. New models of data sharing are needed, in particular when privacy-preserving record linkages (PPRL) are being used to connect data within restricted data enclaves and public open enclaves.

We share a privacy-preserving data-sharing infrastructure that employs a linkage honest broker implementation of PPRL connecting structured electronic health record data between the National Center for Advancing Translational Science (NCATS) National Clinical Cohort Collaborative (N3C), a restricted data enclave, with the National Cancer Institute (NCI) Cancer Imaging Archive (TCIA), a public open enclave.

In particular we will address:

- Data governance models needed when linking data from restricted vs. public enclaves
- Specific privacy-preserving consideration when linking to imaging data which contain specific structured data and imaging considerations
- Privacy-preserving data aggregation when combining multi-modal data from restricted data enclaves with publicly discoverable imaging data

